# Long-term outcomes after surgery to prevent aspiration for patients with amyotrophic lateral sclerosis

**DOI:** 10.1186/s12883-022-02619-z

**Published:** 2022-03-16

**Authors:** Temma Soga, Naoki Suzuki, Kengo Kato, Ai Kawamoto-Hirano, Yuko Kawauchi, Rumiko Izumi, Masaya Toyoshima, Shio Mitsuzawa, Tomomi Shijo, Kensuke Ikeda, Hitoshi Warita, Yukio Katori, Masashi Aoki, Masaaki Kato

**Affiliations:** 1 Department of Neurology, Shodo-kai Southern Tohoku General Hospital, 1-2-5, Satonomori, Iwanuma, Miyagi Japan; 2 Department of Neurology, Tohoku University Graduate School of Medicine, 1-1 Seiryo-machi, Aoba-ku, Sendai 980-8574 Japan; 3 Department of Otolaryngology-Head and Neck Surgery, Tohoku University Graduate School of Medicine, 1-1 Seiryo-machi, Aoba-ku, Sendai Japan; 4 Department of Otolaryngology-Head and Neck Surgery, Tohoku University Hospital, 1-1 Seiryo-machi, Aoba-ku, Sendai 980-8574 Japan

**Keywords:** Word, Amyotrophic lateral sclerosis (ALS), Central-part laryngectomy (CPL), Continuous low-pressure aspiration, Surgery to prevent aspiration, Quality of life (QOL)

## Abstract

**Background:**

Amyotrophic lateral sclerosis (ALS) is a progressive neurodegenerative disease that affects motor neurons selectively. In particular, weakness in respiratory and swallowing muscles occasionally causes aspiration pneumonia and choking, which can be lethal. Surgery to prevent aspiration, which separates the trachea and esophagus, can reduce the associated risks. Central-part laryngectomy (CPL) is a relatively minimally invasive surgery to prevent aspiration. No studies have been conducted on the long-term outcomes of surgery to prevent aspiration in patients with ALS. This case series aimed to determine the long-term outcomes of surgery to prevent aspiration and the use of a continuous low-pressure aspirator in patients with ALS by evaluating the frequency of intratracheal sputum suctions performed per day, intra- and postoperative complications, oral intake data, and satisfaction of patients and their primary caregiver to predict improvement in patients’ quality of life (QOL).

**Methods:**

We report a case series of six patients with ALS who underwent CPL along with tracheostomy to prevent aspiration between January 2015 and November 2018. We evaluated their pre- and postoperative status and administered questionnaires at the time of last admission to the patients and their primary caregivers.

**Results:**

The mean follow-up period after CPL was 33.5 months. Aerophagia was a common postoperative complication. The use of a continuous low-pressure aspirator resulted in reduced frequency of intratracheal sputum suctions. All cases avoided aspiration pneumonia. Oral intake was continued for 2–4 years after the tracheostomy and CPL. The satisfaction levels of the patient and primary caregiver were high.

**Conclusion:**

Our case series suggests that the use of a continuous low-pressure aspirator in patients undergoing CPL improves oral intake and reduces the frequency of intratracheal sputum suctions, which improves the QOL of patients with ALS and their families and caregivers. CPL and continuous low-pressure aspiration should be considered as a management option for ALS with significant bulbar and respiratory muscle weakness/dysfunction.

**Supplementary Information:**

The online version contains supplementary material available at 10.1186/s12883-022-02619-z.

## Background

Amyotrophic lateral sclerosis (ALS) is an intractable neurological disease that selectively affects upper and lower motor neurons. These damages can result in systemic muscle weakness, particularly the respiratory and swallowing muscles [1]. Many patients with ALS experience severe aspiration pneumonia due to deteriorated swallowing function [2]. Aspiration pneumonia or other respiratory failure can cause a lethal event as ALS progresses [3]. Patients with ALS who have undergone tracheostomy are at high risk of pulmonary aspiration [4]. Although these manifestations can be managed by frequently suctioning intratracheal sputum (once per hour), these procedures reduce the patient’s quality of life (QOL) and increase caregiver burden [5]. Surgery to prevent aspiration, which separates the trachea and esophagus, can be considered in addition to tracheostomy. The indications for surgery to prevent aspiration in intractable neurological diseases in Japan are often based on these five aspects: (1) intractable dysphagia; (2) inability to communicate by speech and language with no hope for recovery; (3) a strong desire for oral intake and patient consent; (4) repeated aspiration; and (5) aspiration pneumonia, which can lead to large amounts of sputum that require frequent suctioning, which exhausts the patient and caregiver [4]. In addition to preventing aspiration pneumonia, surgery to prevent aspiration has additional benefits. It can reduce the patient and caregiver burden by reducing intratracheal sputum suctions, low-grade fever and improving nutritional status by alleviating physical exhaustion and feeding difficulty caused by potential aspiration and supporting respiratory function [6]. In contrast, symptoms and transient problems associated with surgery to prevent aspiration include taste and flavor disturbances, constipation, nausea, and the requirement to provide training to caregivers to care for a permanent tracheostomy. It is important to inform patients and their families in advance that surgery to prevent aspiration cannot prevent the loss of swallowing function and that the ability to eat food will be impaired.

In Japan, the incidence of tracheostomy ventilator use in patients with ALS is 21.4–57.7%, which is 10 times higher than in western countries [7–9]. However, the prevalence and awareness regarding surgery to prevent aspiration is low even in cases where it may be indicated; this is because there are only few facilities with sufficient information and experience.

To the best of our knowledge, few studies have examined the long-term effects of surgery to prevent aspiration on the feeding status of patients with ALS [10]. In this study, we investigated the long-term course of patients with ALS after surgery to prevent aspiration. We reported a case series of six patients who underwent central-part laryngectomy (CPL), a relatively minimally invasive procedure.

## Methods

### Participants and data collection

Patients were diagnosed with probable (including laboratory supported) or definite ALS based on the revised El Escorial criteria [11]. Data such as age at diagnosis, site of onset, respiratory function, the frequency of intratracheal sputum suctions the frequency of intratracheal sputum suctions performed per day, intra- and postoperative complications, oral intake status, the revised ALS functional rating scale (ALSFRS-R) [12], and abdominal X-ray were collected for each patient. Each patient’s communication status was assessed on a scale of I to V (Stage I, communicates in sentences; Stage II, communicates with one-word responses only; Stage III, communicates with nonverbal yes/no responses only; Stage IV, occasionally cannot communicate due to uncertain yes/no responses; and Stage V, cannot communicate by any means) [13, 14]. Patients were proposed to undergo CPL which is a relatively minimally invasive procedure of separate trachea from esophagus to prevent aspiration, on the premise of tracheostomy-invasive ventilation (TIV) [6]. At the time of obtaining informed consent, we explained the anticipated adverse events and the benefits of surgery to the patient and caregivers. Anticipated adverse events included loss of speech, wound healing failure, wound infection, aerophagia, and trachea stenosis. In contrast, the anticipated benefits of the surgery are free from the anxiety of aspiration and continuation of oral intake. Continuous low-pressure aspiration is a suction system used for patients undergoing tracheostomy and was developed by Drs. Yamamoto and Tokunaga [15]. Continuous low-pressure aspiration was introduced in all cases because of the frequent suctioning after surgery. The cannula, suction pressure and other conditions were the same. The tracheal cannula with double suction inner and lower holes in the lower part of the cannula and the roller pump-type suction machine should be used together. We proposed this system to reduce the burden on caregivers and to alleviate the patient’s distress caused by frequent suctioning.

### Questionnaire survey

A questionnaire about surgery to prevent aspiration was administered to the patient and primary caregivers (spouses) at the last admission. The questionnaire comprised nine items including CPL decision-making, feelings about intra- and postoperative complications, negative and positive impacts on daily life after CPL. Each item was answered on a 5-point scale (1, strongly disagree, 2, disagree; 3, neutral; 4, agree; 5, strongly agree). There was also a blank section at the end of the questionnaire where the respondent could provide additional comments if desired (see Additional file 1). The questionnaire was designed to reflect the respondents’ frank opinions about decision-making and to investigate their feelings before and after undergoing CPL, the positive or negative changes in their bodies, and the long-term effects.

## Results

The mean age of the six patients was 57.7 years, and the mean age at onset was 51 years (Table 1). CPL was performed at the time of tracheostomy and starting TIV between April 2016 and August 2018, with a mean of 42.7 months after onset and a mean of 33.5 months of follow-up. All patients underwent gastrostomy before CPL, and their nutritional status was well maintained. Regarding respiratory function at the time of surgery, their vital capacity percentage (VC%) ranged from 45.7 to 67.3. A preoperative videofluoroscopic (VF) examination of swallowing revealed liquid aspiration in cases 3 and 6. At the time of the study, the longest postoperative period was 60 months and that where patients were still able to retain oral intake was 48 months. Communication status at the time of the survey was IV and V in cases 3 and 6, respectively, whereas others could communicate using a computer [13].

**Table 1:** Case profile and outcome of surgery to prevent aspiration and continuous low-pressure aspiration

	Case 1	Case 2	Case 3	Case 4	Case 5	Case 6	Average
Age at last admission/sex	56/M	48/F	46/F	49/M	63/M	68/M	57.7
First ALS symptom	Lower limb	Upper limb	Upper limb	Bulbar	Lower limb	Bulbar	
Duration from symptom onset to CPL (months)	22	42	48	25	34	17	42.7
Duration after CPL (months)	55	37	24	39	22	60	33.5
VC % at CPL	45.7	NA	47.5	67.3	64.4	NA	
FVC % at CPL	50.9	NA	61.9	58.6	58.3	NA	
Aspiration with water	–	–	+	–	–	+	
Status of oral intake at the examination	1 meal oral intake	2 meals oral intake	2 meals oral intake	Limited smallest oral intake	1 meal oral intake	No oral intake	
Aspiration pneumonia after CPL	None	None	None	None	None	None	
Number of sputum suctions after CPL	12–20	12–20	12–15	12–20	20	NA	
Timing of introduction of continuous low-pressure aspiration after CPL (months)	3	1	2	2	0.5	No use	1.7
Number of sputum suction after the introduction of continuous low-pressure aspiration	0–1	0–1	0–1	3–4	10–12	No use	
Outcome of the use of continuous low-pressure aspiration	Good	Good	Good	Good	Good	NA	
Aerophagia after CPL	+	+	+	–	+	+	
Communication status	I	I	IV	I	I	V	
ALSFRS-R score at the last admission	1	2	2	1	4	0	

*Abbreviations*: *CPL* central-part laryngectomy, *VC%* vital capacity percentage, *FVC%* percent of predicted forced vital capacity, *ALSFRS-R* the revised amyotrophic lateral sclerosis functional rating scale

**Fig. 1:**
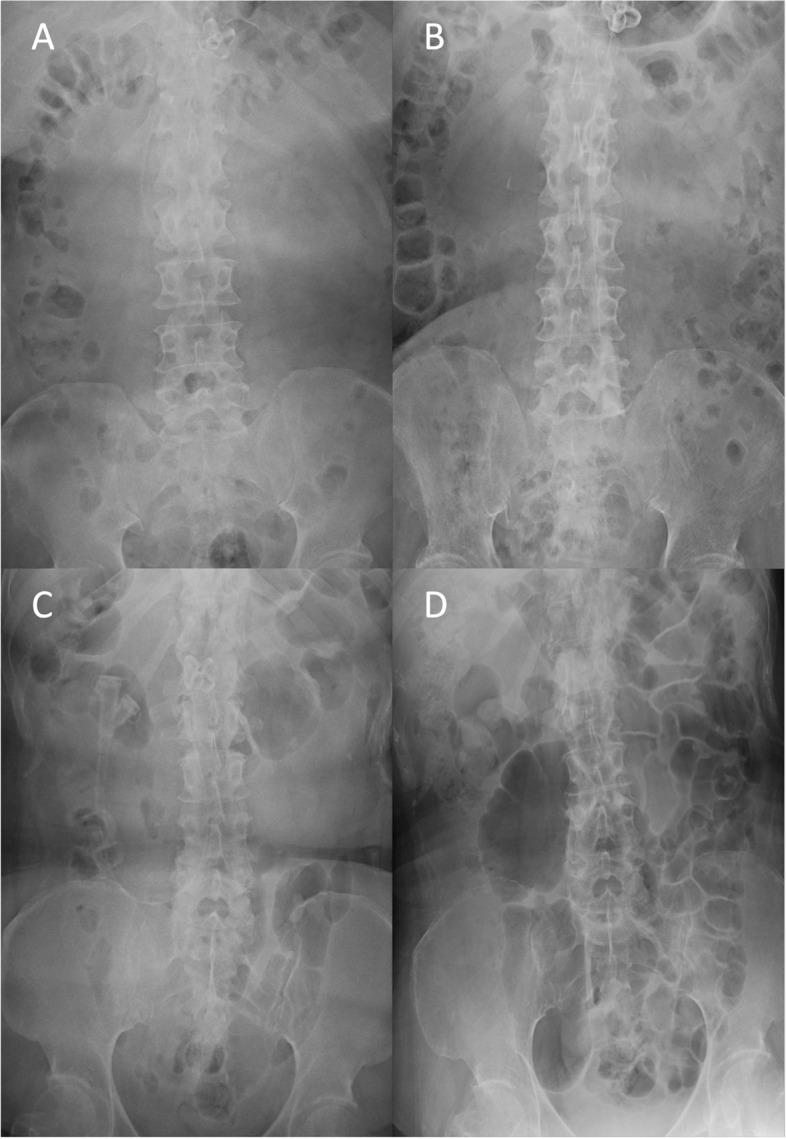
Abdominal X-ray in representative cases. **A** is an abdominal X-ray of Case 4 before central-part laryngectomy (CPL) showing the usual amount of intestinal gas; **B** is an abdominal X-ray of Case 4 20 months after CPL still showing the usual amount; **C** is an abdominal X-ray of Case 2 before CPL showing the usual amount of intestinal gas; **D** is an abdominal X-ray of Case 2 30 months after CPL with more than the usual amount of intestinal gas indicating evidence of aerophagia

The immediate postoperative course was confirmed for five of the six cases. The immediate postoperative complication was aerophagia in four cases. Although the degree of subjective symptoms varied, abdominal radiographs showed gastric and intestinal gas retention in all four cases (Fig. 1). This finding persisted at follow-up and worsened with continued feeding. Symptoms tended to be relieved approximately 1 month postoperatively. We were able to alleviate subjective symptoms by performing active supine positioning, degassing before meals using the gastric tube, continuous suctioning in the mouth except during meals, and swallowing training. In patients receiving continuous oral intake, gastric and intestinal gas retention persisted for a long period (Fig. 1C and D). However, intestinal gas retention was not observed in patients with little or no oral intake (Fig. 1A and B). After CPL, five cases required sputum suctioning 15–20 times a day; however, introducing a continuous low-pressure aspirator through the inner cannula suction hole resulted in the frequency of intratracheal sputum suctions decreasing to 1–4 times a day, except for case 5 (Table 1).

One patient (Case 6) with communication difficulties voluntarily stopped eating, whereas all other patients continued oral intake with the assistance of their primary caregiver. In four cases, we obtained the questionnaire responses directly from the patients or with help from their caregiver. The remaining patients had difficulty communicating and could not respond. We obtained responses from the primary caregivers for all six cases (Table 2). The mean score for “Are you glad you or your primary caregivers did surgery to prevent aspiration?” was 4.00 for the patients and 4.40 for the primary caregivers, indicating that both the patient and caregiver, especially caregivers, were highly satisfied with the long-term course of the procedure to prevent aspiration. For having sufficient information about surgery to prevent aspiration, the mean score of the patient was 4.50 and of the primary caregiver was 3.83. As the patient was already aware of the information provided regarding the surgery, there were no major unanticipated adverse events after CPL. In contrast, for the “minor adverse events” category, two patients commented on the postoperative change in taste and aerophagia. Free comments about changes after surgery to prevent aspiration included “In addition to the reassurance of not aspirating, the mental peace of being able to take food orally” (Case 1), “Eating out with my family has become a way of life” (Case 2), and “I can now smell food” (Case 2).

**Table 2:** Questionnaire items

No.	Questions	Patient/mean score	Caregiver/mean score
1	Were you proactive to surgery to prevent aspiration?	4.5	3.2
2	Are you glad you or your primary caregivers did surgery to prevent aspiration?	4	4.4
3	Have you been able to adequately talk to others about surgery to prevent aspiration?	4	3.7
4	Do you feel that you have been provided with enough information about surgery to prevent aspiration?	4.5	3.8
5	Have there been any unanticipated serious adverse events following surgery to prevent aspiration?	1.5	1.8
6	Have there been any unanticipated small adverse events after surgery to prevent aspiration?	3.25	3
7	Have there been any unanticipated significant good things about surgery to prevent aspiration?	3.75	3.5
8	Have there been any unanticipated small good things about surgery to prevent aspiration?	3	3
9	Do you think there has been any long-term change after surgery to prevent aspiration?	4.25	2.8

## Discussion

### CPL for patients with ALS

Surgery to prevent aspiration mainly includes total laryngectomy, laryngotracheal dissection, and tracheoesophageal anastomosis [4]. Tracheoesophageal anastomosis was described by Lindeman in 1975 [16] and its variant, laryngotracheal dissection, was described in 1976 [17]. Total laryngectomy is a common procedure for laryngeal cancer, and many surgeons perform it. However, the surgical invasiveness of laryngectomy is greater than that of other techniques; this can be a problem for patients with ALS with advanced respiratory impairment. Although tracheoesophageal anastomosis is anatomically reversible in terms of vocal function, total laryngectomy is irreversible. This situation is not a problem for patients with ALS due to the progressive course of the disease.

In contrast, CPL is less invasive than total laryngectomy because of its smaller resection area. Removal of the cricoid cartilage and amputation of the cricopharyngeal muscle creates a wider laryngeal cavity, improving the patient’s swallowing ability [6]. The six patients in this study needed long-term TIV management. We chose to perform CPL as it provides a superior fixation of the tracheal cannula and an appropriate location for the tracheal hole that does not interfere with swallowing. To retain oral intake without concerns about the onset of aspiration pneumonia, we performed TIV and CPL simultaneously. The patient had no trouble undergoing the surgical procedure. The purpose of CPL was to preserve the function of eating. The patients should have preserved function and show a will to eat and drink food before taking the decision to perform CPL. Therefore, the timing of CPL should be earlier than when it is performed to solely avoid aspiration.

### Aerophagia as a common postoperative complication

Some of the complications after surgery to prevent aspiration are worsening pneumonia, wound pain, intraoperative bleeding, wound healing failure, wound infection, aerophagia, and postoperative trachea stenosis. Although no intraoperative complications were observed, five of six patients had aerophagia as a postoperative complication. In this study, aerophagia was the most common complication during the early postoperative period. No intestinal gas retention was reported in patients with reduced oral swallowing in the state of no eating or only enjoying the taste of jelly-like food. This result raises the possibility that aerophagia may be related to the preservation of swallowing function. Aerophagia is assumed to be a result of the reduced resistance to swallowing from the hypopharynx to the cervical esophageal inlet due to cricopharyngeal chondrotomy and cricopharyngeal muscle transection by CPL. In addition, the loss of oral function associated with ALS makes it difficult to separate the food mass from air during swallowing. However, this could be a byproduct of the advantage of preserving the swallowing function via CPL.

Conversely, even in patients with a long-term course, continuous degassing via gastrostomy before and after meals helped support continued eating. There were no cases of repeated vomiting associated with abdominal distension. Although aerophagia is a common complication, it is manageable. Early intervention for aerophagia is recommended in cases where patients have a strong desire to continue feeding by performing surgery to prevent aspiration.

### Continuous low-pressure aspiration reduces the caregiver burden

It is said that the frequency of intratracheal sputum suction can be reduced in patients after surgery to prevent aspiration [18], but for the cases in this study, the frequency of intratracheal sputum suctions per day after surgery was high, 20, which is still a great burden for caregivers in the home care setting. For the five cases in this study, introducing a continuous low-pressure aspirator reduced the frequency of sputum suctioning. It is assumed that continuous low-pressure aspiration can greatly reduce the burden on patients and caregivers [15]. The cost of introducing this equipment is high, and the caregiver needs training to manage tube obstructions, but it is worth a try.

In the case of tracheostomy plus continuous low-pressure aspiration, continuous low-pressure aspiration reduces the caregivers’ burden but cannot reduce the risk of aspiration/choking. Surgery to prevent aspiration, including CPL, reduces aspiration/choking anxiety. For those who want to eat orally, avoid aspiration/choking, and reduce caregivers’ burden, we propose the combination of continuous low-pressure aspiration and CPL.

### QOL

In this study, we included cases who wanted to continue feeding and receive TIV for decreased respiratory function (VC 45.7–67.3%) and were not observed to have severe dysphagia on VF. The questionnaire survey results revealed that the patients and their primary caregivers were satisfied with the outcomes of surgery to prevent aspiration in general. However, there was a large discrepancy between the patients’ and primary caregivers; response regarding “Were you proactive to surgery to prevent aspiration?” This discrepancy may reflect the caregiver’s anxiety rather than the patient’s wishes. Knowing that the primary caregiver is satisfied with long-term outcome of surgery to prevent aspiration can help to resolve the discrepancy between the patient’s wishes and those of the primary caregiver.

It has been reported that patients with ALS maintain a positive sense of well-being even after receiving life-prolonging treatment via ventilator or gastrostomy or suffer from a completely locked-in state [19]. The patients in this study underwent gastrostomy and tracheostomy, aspiration prevention, prolongation of the feeding period, and continuation of feeding. The intervention for most of the patients in this study was made before the stage of aspiration on VF, and feeding was continued for up to 55 months. The prospect of a long-term continuation of feeding in exchange for earlier loss of speech should be considered when evaluating surgery to prevent aspiration as an option for improving the patient’s QOL. Establishing the communication method before the patient undergoes surgery is necessary to prevent aspiration. Surgery to prevent aspiration reduces aspiration anxiety, sputum suctioning, and the burden on caregivers; some patients considered continued oral intake to be their hope for life as it improved their QOL.

### Limitation

One limitation of this study was the small number of cases as only four of the six patients could respond to the questionnaire; thus, we could not compare any control group. The longest period was 60 months. It is necessary to continue the evaluation process over a longer period to determine long-term postoperative outcomes. We could not determine the optimal time point for performing CPL because of the small number of cases and the short observation period.

In this study, we administered a questionnaire to the patient and spouse. Opinions from caregivers other than the spouse should be considered [19]. Continuing oral intake for a long time after a prolonged disease can improve the QOL, but it can also become a disadvantage to caregivers as it consumes time and labor. In some cases, instead of eating, the patients should undergo physical therapy to avoid contractures. There are cases in which patients may become fixated on oral intake due to a decline in frontal dysfunction. Practically, oral intake will be available only within 1–2 years after the surgery. It is important to provide sufficient information to both the patient and caregivers before the surgery.

## Conclusions

We report the long-term outcomes of CPL for ALS. We believe that continuous low-pressure aspiration in the airway after surgery to prevent aspiration is useful to maintain oral intake and reduce the frequency of intratracheal sputum suctioning, thereby improving the QOL of patients and their families.

## Supplementary Information

**Additional file 1.** Questionnaire, Detail of the questionnaire for patients with ALS after surgery to prevent aspiration and for primary caregivers.

## Data Availability

The datasets during and/or analyzed in the current study are available from the corresponding author upon reasonable request.

## Abbreviations

ALS: Amyotrophic lateral sclerosis
ALSFRS-R: the revised ALS functional rating scale
CPL: Central-part laryngectomy
QOL: Quality of life
TIV: Tracheostomy-invasive ventilation
VC%: Vital capacity percentage
VF: Videofluoroscopic examination of swallowing
