# Supplementary material for: Long-term outcomes after surgery to prevent aspiration for patients with amyotrophic lateral sclerosis
Source: BMC Neurol. 2022 Mar 16;22:94. doi: 10.1186/s12883-022-02619-z (PMC8925201; doi:10.1186/s12883-022-02619-z)
Supplement: Supplementary file 1 — Additional file 1. Questionnaire, Detail of the questionnaire for patients with ALS after surgery to prevent aspiration and for primary caregivers. [file 12883_2022_2619_MOESM1_ESM.docx]

Questionnaire for patients with ALS after surgery to prevent aspiration and for primary caregivers

Were you proactive to surgery to prevent aspiration?

1, strongly disagree; 2, disagree; 3, neutral; 4, agree; 5, strongly agree

Additional comments ( )

Are you glad you or your primary caregivers did surgery to prevent aspiration?

1, strongly disagree; 2, disagree; 3, neutral; 4, agree; 5, strongly agree

Additional comments ( )

Have you been able to adequately talk to others about surgery to prevent aspiration?

1, strongly disagree; 2, disagree; 3, neutral; 4, agree; 5, strongly agree

Additional comments ( )

Do you feel that you have been provided with enough information about surgery to prevent aspiration?

1, strongly disagree; 2, disagree; 3, neutral; 4, agree; 5, strongly agree

Additional comments ( )

Have there been any unanticipated serious adverse events following surgery to prevent aspiration?

1, strongly disagree; 2, disagree; 3, neutral; 4, agree; 5, strongly agree

Additional comments ( )

Have there been any unanticipated small adverse events after surgery to prevent aspiration?

1, strongly disagree; 2, disagree; 3, neutral; 4, agree; 5, strongly agree

Additional comments ( )

Have there been any unanticipated significant good things about surgery to prevent aspiration?

1, strongly disagree; 2, disagree; 3, neutral; 4, agree; 5, strongly agree

Additional comments ( )

Have there been any unanticipated small good things about surgery to prevent aspiration?

1, strongly disagree; 2, disagree; 3, neutral; 4, agree; 5, strongly agree

Additional comments ( )

Do you think there has been any long-term change after surgery to prevent aspiration?

1, strongly disagree; 2, disagree; 3, neutral; 4, agree; 5, strongly agree

Additional comments ( )

If you have any comment about surgery to prevent aspiration, please write here. (e.g., messages to people who considering surgery to prevent aspiration, requests to medical staffs)
